# Effect of Injectable Platelet-Rich Fibrin with a Nano-Hydroxyapatite Bone Graft on the Treatment of a Grade II Furcation Defect

**DOI:** 10.3390/bioengineering9110602

**Published:** 2022-10-22

**Authors:** Uma P. Nair, Ravindra Shivamurthy, Raghavendra Reddy Nagate, Saurabh Chaturvedi, Saad M. Al-Qahtani, Mohammad Al Magbol, Shankar T. Gokhale, Shreyas Tikare, Mudita Chaturvedi

**Affiliations:** 1 Department of Periodontology, JSS Dental College & Hospital, Mysuru 570015, India; 2 Department of Periodontics and Community Dental Sciences, College of Dentistry, King Khalid University, Abha 61421, Saudi Arabia; 3 Department of Prosthetic Dentistry, College of Dentistry, King Khalid University, Abha 61421, Saudi Arabia; 4 Multispecialty Dental Clinic, Bhopal 462026, India

**Keywords:** furcation defect, bone graft, periodontal guided tissue, platelet-rich fibrin

## Abstract

**Background**: Periodontal diseases lead to bone loss, crestal defects and even loss of the tooth, which also further makes it difficult to replace the tooth. Autogenous bone grafts are considered the gold standard in bone regenerative procedures. This study aimed to compare and evaluate the bone regenerative effects of i-PRF (Injectable- Platelet-rich fibrin) with a bone graft and a bone graft alone in mandibular grade II furcation defects over a period of 9 months. **Method**: This was a comparative study of 12 participants, who were randomly selected and grouped into two groups: test and control. Following phase I therapy, both groups were subjected to open flap debridement. In the test group, after debridement, a nano-hydroxyapatite bone graft mixed with i-PRF was inserted, whereas in the control group only a nano-hydroxyapatite bone graft was inserted. The clinical parameters such as plaque index (PI), gingival index (GI), pocket probing depth (PPD), clinical attachment level (CAL), horizontal probing depth (HPD) and vertical probing depth (VPD) were recorded at baseline, 3 months, 6 months and 9 months following the surgery. The bone area fill (BAF) was assessed using intraoral periapical radiographs (IOPARs) taken at baseline and 9 months after surgery. **Results**: At the baseline, there was no statistically significant difference between the tested parameters. After 9 months all the clinical parameters, PI, GI, PPD, CAL, HPD and VPD as well as radiographic bone fill showed a significant increase in both the groups (*p* < 0.05) (PI-TGr; CGr–VPD—3.5 ± 0.54 to 0.66 ± 0.51; 3.3 ± 0.81 to 2 ± 0.63/BAF—2.9 ± 0.88 to 5.6 ± 1.10; 3.4 ± 1.39 to 3.9 ± 1.4). On comparison the test group showed better results for each clinical parameter. **Conclusion**: The results showed increased improvement in clinical conditions in both groups, although better results were seen in the group where i-PRF with a nano-HA bone graft was used in the furcation defect.

## 1. Introduction

Periodontitis is an inflammatory condition of the teeth and the supporting structure which leads to attachment loss as well as the loss of bone which eventually leads to tooth loss. Multirooted teeth pose a special challenge to the clinician due to the inaccessibility of the furcation involved [1]. The oral cavity is continuously exposed to the action of endogenous and exogenous biological, chemical and physical factors that are responsible for the excessive production of reactive oxygen species, resulting in periodontitis [2]. Various surgical and non-surgical treatments have been described in the literature such as laser or antimicrobial photodynamic therapy, also combined with non-surgical periodontal treatment, and systemic treatment with melatonin provides additional improvements to severe periodontal conditions [3,4]. Moreover, sonic and ultrasonic devices are effective in disrupting the biofilm and carefully removing soft and hard deposits from a root surface with minimal trauma to the tooth structure [5].

Autogenous bone grafts are considered the gold standard in bone regenerative procedures. Chitsazi et al. [6] conducted a study where they compared a nano-hydroxyapatite bone graft with an autogenous bone graft in the treatment of an intrabony defect. The results showed that the nano-hydroxyapatite bone graft had regenerative effects comparable to those seen in autogenous bone grafts enabling them to be a suitable substitute.

Various treatment modalities have been adopted for the treatment of furcation-involved teeth which include scaling and root planing, open flap debridement, regenerative procedures such as guided tissue regeneration, guided bone regeneration, platelet concentrates, hemisection, root resection and extraction in terminal cases [7,8].

Regenerative procedures have gained popularity in the utilization of the platelet concentrates mixed with bone grafts. Platelet concentrates such as platelet-rich plasma and platelet-rich fibrin have been widely used in intra-bony defects. Platelets are a good source of growth factors and leukocytes which facilitates tissue regeneration and better wound healing. First-generation platelet concentrates, platelet-rich plasma (PRP), is a liquid formulation which is prepared by adding anti-coagulants into the blood samples before subjecting it to centrifugation. It has been used in the field of medicine for a long time. However, the addition of the anti-coagulants causes delayed healing [9].

Then the second-generation platelet concentrates, platelet-rich fibrin (PRF), emerged which do not use any additives. It is prepared by collecting the blood in a glass tube and immediately centrifuging it. This results in the formation of a coagulum which is used as such or converted into a membrane by applying pressure to the coagulum in a PRF box [9]. Various modifications to the centrifugation process resulted in the development of different forms of platelet concentrate such as concentrated growth factor (CGF), advanced platelet-rich fibrin (A-PRF) and titanium platelet-rich fibrin (T-PRF). However, one of the drawbacks for all these is that they all are in the form of coagulum/gel form and are not conducive to being injected which has limited the application of these products [10,11].

This resulted in the development of the injectable form of platelet-rich fibrin (i-PRF) which is formed by decreasing the centrifugation speed and time. It is prepared using a low-speed centrifugation concept (LSCC) which includes the centrifugation of the blood sample at 700 rpm for 3 min. It has been proposed that for i-PRF the initial separation of the blood components happens in the first 2–4 min. Moreover, by reducing the centrifugation speed and time, more platelets and growth factors are retained in the supernatant fluid before the coagulation cascade is initiated [12,13].

Studies have proven that a bone graft when used with platelet concentrates yields better regeneration in intra-bony defects. PRP, PRF and bone grafts with PRF membranes have been shown to give better results as compared to bone graft alone [14], but still, the consistency in the results of studies in the literature is missing. Moreover, very few studies have been conducted where bone grafts were mixed with i-PRF in furcation defects and also the results were not consistent. Thus, owing to these facts the present randomized clinical trial was conducted with the aim to compare and evaluate the bone regenerative effects of i-PRF with bone graft and bone graft alone in mandibular grade II furcation defects over a period of 9 months both clinically and radiographically. The null hypothesis formulated was that there will be no variation in the healing of mandibular grade II furcation defects treated with i-PRF with bone graft and bone graft alone.

## 2. Materials and Methods

### 2.1. Study Design

The sample size estimation was performed using the formula S = $\frac{Z^{2}pq}{d^{2}}$ ^=^$\;\frac{1.96\times 1.96\times 0.005\times 0.995}{0.05\times 0.05}$. The sample size was estimated to be minimum 8 patients per group. (Where S is the sample size, *Z* is the statistic corresponding to the level of confidence, *p* is expected prevalence (obtained from the pilot study), *q* is (1 − *p*) and *d* is precision (corresponding to effect size)) owing to availability of patients and time we recruited total 16 patients per group. Thus, in this interventional study a total of 32 patients were selected from the dental outpatient department of the Department of Periodontology. All the patients who fulfilled the inclusion criteria were selected and grouped into two groups by lottery method. Group-I Control group–Open flap debridement with bone graft was performed; and Group-II test group–Open flap debridement with sticky bone (i-PRF + bone graft) was performed.

Ethical approval was obtained from the Institutional Ethical Committee of the institution (Research protocol No: 09/2018). (JSS Dental College and Hospital, Mysuru, India). Written consent was obtained from the patients before the commencement of the study. The inclusion criteria were: 1. Systemically healthy subjects between the age group of 30 and 55 years both male and female; 2. Clinical and radiographic evidence of mandibular buccal furcation defect ≥ 3 mm; 3. Vital tooth (Cold test with endo-ice {Coltène CO-H05032 Hygenic Endo Ice Spray, Coltene/Whaledent Inc., Cuyahoga Falls, OH, USA}); 4. Vertical probing depth ≥ 5 mm, Horizontal probing depth ≥ 3 mm. (Glickman’s classification of furcation involvement for horizontal, Tarnow and Fletcher classification for the vertical component of furcation) [15,16]. The exclusion criteria were: 1. Subjects using tobacco in any form; 2. Pregnant or lactating mother; 3. Known allergy to any of the materials; 4. Subjects on any medication; 5. Subjects on antibiotic therapy within the last 3 months; 6. subjects with malocclusion; 7. Subject with the diagnosis of pulpo periodontal lesion; 8. Any other occlusal discrepancy; 9. Diabetic patients. Clinical parameters such as plaque index (PI), gingival index (GI), pocket probing depth (PPD), clinical attachment level (CAL), horizontal probing depth (HPD) and vertical probing depth (VPD) were recorded at baseline, 3 months, 6 months and 9 months [17]. Radiographs were taken with the grid at baseline and 9 months postoperatively and bone area fill (BAF) was measured. The conventional intraoral periapical radiographs were scanned using a transparency scanner. Radiographic assessment of horizontal and vertical bone fill in the area of furcation was completed using AutoCAD software version 2021 with the assistance of trained computer personnel. To standardize the horizontal probing depth, a modified stent made of clear acrylic resin was used. The modification was in terms of the extension of the buccal plate up to the attached gingiva to go beyond the furcal entrance. A hole was made at the buccal extension of the stent approximately coinciding with the furcal entrance to guide the probe penetration (Naber’s probe) in the same direction. The clinical assessment was completed using a Naber’s probe and radiographically by conventional intraoral periapical radiographs (IOPAR) with X-ray mesh gauge (grid) using the paralleling technique. The Rinn xcp (XCP stands for “extension cone paralleling”) (Dentsply Sirona Charlotte, Charlotte, NC, USA) was used in each patient while shooting radiographs, so as to standardize the radiograph shooting.

### 2.2. I-Prf Preparation Method

i-PRF was prepared by Choukroun’s method. A total of 10 mL of venous blood was drawn from the patient and was immediately centrifuged for 3 min at 700 rpm in the laboratory centrifuge (Labtech-Dentifuge). The orange-colored liquid supernatant obtained after centrifugation was collected using a syringe. A limited amount of i-PRF was obtained from 10 mL of blood sample (1.5 mL approximately).

### 2.3. Surgical Procedure

**Figure 1:**
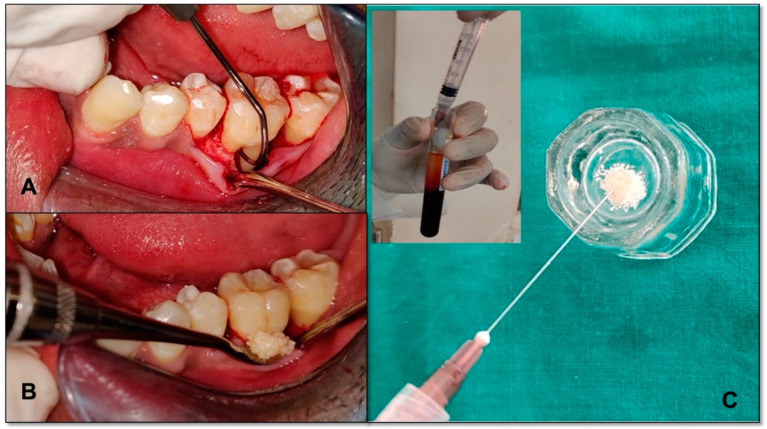
(**A**) Grade II furcation involvement; (**B**) i-PRF collection from the test tube (insight) and mixed with nano-hydroxyapatite bone graft; (**C**) nano-hydroxyapatite bone graft mixed with i-PRF placed into the defect.

**Figure 2:**
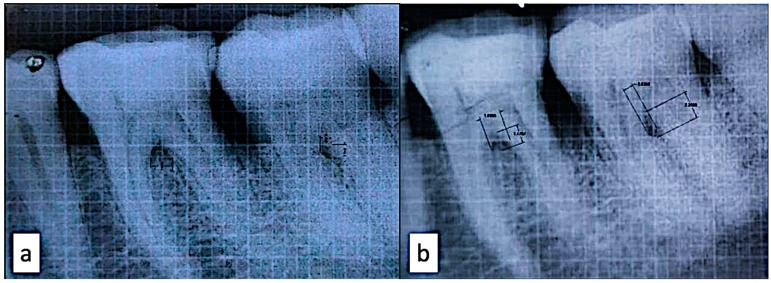
(**a**) IOPAR at baseline; (**b**) IOPAR at 9 months.

After phase I therapy, the full-thickness mucoperiosteal flap was raised and thorough debridement was completed. In the mandibular grade II furcation defect of the control group, the nano-hydroxyapatite bone graft (Sybograft, Eucare pharmaceutical pvt LTD. Chennai, India) alone and in the test group, the nano-hydroxyapatite bone graft mixed with i-PRF (0.5 gms nano-hydroxyapatite bone graft mixed with 1.5 mL i-PRF) were inserted (Figure 1). The flaps were approximated using non-resorbable silk sutures with a vertical mattress suturing technique. The full-thickness envelope flap was employed to prevent any kind of perforation of the flap leading to the spilling of the graft material from the bony defect area. The periodontal dressing was inserted. Post-operative instructions and Amoxycillin 500 mg (Amoxycillin 500 mg was advised three times daily for 5 days). The patients were recalled after 1 week for suture removal. They were recalled at 3 months, 6 months and 9 months postoperatively for check-ups and for the recording of the clinical parameters. The radiograph was taken once at the baseline and the second 9 months post-operatively (Figure 2a,b). The patients were informed about the healing process and the time involved. It was also explained that the oral cavity hosts a plethora of tissues that serve various functions, and in this complex environment cells, bacteria, viruses and fungi live in a subtly balanced ecosystem [18]. It is this complexity of factors, both systemic and local, that offer the healing particularities and challenges of the oral cavity. In case of any healing issues, the patient could relate the process and report the problem to the chief researcher and/or any team member.

### 2.4. Statistical Analysis

The statistical analysis was performed using SPSS software version 22. Within- group comparisons for each group at different time intervals were completed by paired sample *t*-test. Between- group comparisons at different time intervals were performed by an independent sample *t*-test and repeated measures ANOVA.

## 3. Results

For all the participants PI, GI, PPD, CAL, HPD and VPD were recorded at baseline, 3 months, 6 months and 9 months. Table 1 shows the demographic data of the study. The comparison was performed for the tested parameters at baseline between the control and the test group and no statistically significant difference was noted in all parameters (Table 2). Radiographic bone area fill was recorded at baseline and 9 months. A total of 24 participants completed the study, with 12 participants in each group. During the course of the study, six patients opted out of the study and two patients retracted their consent, so we excluded them from the study samples. The mean age of participants was 35.2 years.

### 3.1. Plaque Index (Pi)

The mean plaque index of the test group reduced from 1.4 ± 0.28 at baseline to 0.44 ± 0.16 at 9 months. The mean PI of the control group reduced from 1.2 ± 0.12 at baseline to 0.5 ± 0.22 at 9 months. When compared between the groups, no statistically significant difference was noted indicating that both groups satisfactorily maintained oral hygiene. When compared within each group, there was a statistically significant difference by the end of 9 months (*p* = 0.334) (Table 3).

### 3.2. Gingival Index

The mean GI score of the test group reduced from 1.24 ± 0.25 at baseline to 0.3 ± 0.15. The mean GI score of the control group reduced from 1.2 ± 0.14 at baseline to 0.5 ± 0.32. When compared between the groups, no statistically significant difference was noted indicating that both groups satisfactorily maintained oral hygiene. When compared within each group, there was a statistically significant difference by the end of 9 months (*p* = 0.140) (Table 3).

### 3.3. Pocket Probing Depth

The mean PPD of the test group reduced from 6.0 ± 0.89 at baseline to 1.3 ± 0.51 at 9 months. The mean PPD of the control group reduced from 5.5 ± 1.22 at baseline to 2.1 ± 0.40 at 9 months. When compared between the groups, a statistically significant difference favoring the test group was noted. When compared within the group, there was a statistically significant reduction in both groups by 9 months (Table 3).

### 3.4. Clinical Attachment Level

The mean CAL of the test group improved from 5.1 ± 0.75 at baseline to 1.0 ± 0.0 at 9 months. The mean CAL of the control group was from 3.8 ± 1.83 at baseline to 1.3 ± 0.51 at 9 months. When compared between the groups, a statistically significant difference favoring the test group was noted. When compared within the group, a statistically significant improvement was seen in both groups (Table 3).

### 3.5. Horizontal Probing Depth

The mean HPD of the test group reduced from 4.0 ± 1.26 at baseline to 0.66 ± 0.51 at 9 months. The mean HPD in the control group reduced from 4.3 ± 1.03 at baseline to 2.6 ± 1.21 at 9 months. When compared between the groups, a statistically significant difference was noted favoring the test group. When compared within the group, a statistically significant reduction was noted in both the groups at the end of 9 months (Table 3).

### 3.6. Vertical Probing Depth

The mean VPD of the test group reduced from 3.5 ± 0.54 at baseline to 0.66 ± 0.51 at 9 months. The mean VPD of the control group reduced from 3.3 ± 0.81 at baseline to 2 ± 0.63 at 9 months. When compared between the groups, a statistically significant difference was noted favoring the test group. When compared within the group a statistically significant reduction was seen in both the groups by 9 months (Table 3).

### 3.7. Bone Area Fill

The mean BAF of the test group improved from 2.9 ± 0.88 at baseline and 5.6 ± 1.10 at 9 months. The mean BAF of the control groups improved from 3.4 ± 1.39 at baseline to 3.9 ± 1.4 at 9 months. When compared between the groups, a statistically significant difference was noted in favor of test group. When compared within the group, a statistically significant improvement was noted in both the groups (Table 3).

## 4. Discussion

In the last four decades, periodontal therapy has seen significant progress in various aspects. There has been a complete shift from resective pocket eradicative procedures to techniques and methods aimed at the regeneration and conservation of the periodontium. Bone, being the crux of periodontal disease, has obviously received a lot of attention. A number of bone grafts have been used with or without the use of i-PRF in the treatment of various osseous defects such as vertical defects and furcation defects in different clinical trials with different degrees of success. Upon analysis of the various clinical trial research results, it was seen that most of the bone graft materials such as DFDBA, FDBA, Hydroxyapatite, Ceramics, etc., when used with or without i-PRF resulted in significant improvement in the clinical parameters. Along with this, the suturing technique had an important role, although it was described that silk sutures have a high rate of bacterial adherence [19] in the present study it was taken care that this would not affect the outcome by using the vertical mattress suturing technique so as to avoid the contact of the suture material with graft material and i-Prf.

However, the amount of bone fill, the type of bone tissue that has formed and the exact nature of healing have not been consistent. Many of these bone grafts have been found to be no more than fillers of bone defects without demonstrating any osteogenic or osteoconductive potential until the advent of i-PRF which showed significant improvements when used along with bone grafts.

In the present study, i-PRF with nano-hydroxyapatite (nano-HA) bone graft was used as the test while nano-hydroxyapatite bone graft alone was used as the control in the treatment of mandibular grade II furcation defect. As per the literature survey, there are no studies that have reported the use of autologous i-PRF in the treatment of furcation defects. A clinically and statistically significant improvement in the PPD, CAL, HPD, VPD and radiographic bone area fill was noted in both the groups at 9 months postoperatively compared to baseline and the null hypothesis formulated was rejected. Hydroxyapatite is a calcium phosphate compound which is the most stable and least soluble form found in nature when compared to other forms of calcium phosphate [20]. It has good biocompatibility and bioactivity as it mimics the inorganic substance of the bone. The only disadvantage is the porous structure of this substance. Singh VP et al. conducted a study to evaluate the efficacy of nano-HA bone graft along with bioresorbable collagen membrane when compared to open flap debridement [21].

The results showed improvement in the group where the nano-HA bone graft with the collagen membrane was used. This study was performed using only the nano-HA bone graft and it was seen that it yielded better results when compared to open flap debridement. Autogenous bone grafts are considered the gold standard in bone regenerative procedures. A study was performed by Chitsazi et al. [5] to compare the regenerative potential of autogenous bone graft (ABG) and nanocrystalline hydroxyapatite in intrabony defects. It showed that nano-HA bone graft had regenerative effects similar to that of an autogenous bone graft thus proving to be a suitable replacement for the autogenous bone graft. Another property of nano-HA is its antibacterial and anti-inflammatory effect as it is a modulator for monocytes and macrophages [22]. When embedded in a suitable scaffold, it can be successfully used in regenerative procedures for periodontal bone defects. These scaffolds can either be ceramic scaffolds or biologic scaffolds such as PRF, polymer or, in this case, i-PRF. Recently, since platelet concentrates started gaining popularity, studies have been performed to compare their regenerative effects when used alone and along with bone grafts. A study was completed by Mathur et al. where comparison was completed between the regenerative effect of PRF and Autologous bone graft (ABG) when placed in intrabony defects. The results showed that increased attachment gain was noted in the PRF group [23]. Mohamad Fouad Edrees et al. completed a study to evaluate the effect of platelet rich fibrin combined with nano-hydroxyapatite bone graft in mandibular grade II furcation as opposed to the bone graft alone. The results showed increased attachment gain in the test group with PRF [24]. Similar studies were performed by Pradeep et al. [25], Attia AM [26], Elgendy and Abo [27] and Siddiqui ZR [28] where improved regeneration and bone fill was noted when PRF was used along with the bone graft. Platelets are proven to be a good source of growth factors and all the above-mentioned studies showed that second-generation platelet concentrate, PRF, with bone graft had a superior effect when compared to bone graft alone. However, when compared to PRP, one drawback that limits the applications of PRF is that it is obtained in a gel form and hence it is not conducive to being injected [29]. To overcome this, an injectable form of the PRF was recently developed, which was termed injectable-PRF (i-PRF). Studies have shown that i-PRF is rich in growth factors, antimicrobial proteins, complement binding proteins and antimicrobial peptides [30,31], and has a regenerative effect as well. It was used to increase the thickness of the gingiva, through neoangiogenesis and neocollagenesis [32]. It was also used for local drug delivery and for root biomodification during root coverage procedures [33,34]. The ability of i-PRF to bind to the bone grafts as a platelet aggregate for bone regeneration facilitated the use of i-PRF as an alternative to PRP [35]. Kour P et al. completed a study to compare the antimicrobial efficacy of PRP, PRF and i-PRF against Porphyromonas gingivalis (Pg) and Aggregatibacter actinomycetemcomitans (Aa). Results showed that i-PRF had a wide zone of inhibition for Pg while PRP had a wide zone of inhibition against Aa [36]. Wang et al. conducted a study evaluating the effects of i-PRF where it was concluded that i-PRF was able to substantially influence the migration, proliferation and differentiation of human osteoblasts as compared to PRP [37]. Kyyak et al. completed a study to compare an allogenic (ABSM) and a xenogenic bone substitute material (XBSM) with and without injectable platelet-rich fibrin on cell characteristics of osteoblasts. Results showed that i-PRF with ABSM increases the osteoblast activity as compared to XBSM with i-PRF or untreated bone substitute in vitro [38]. Raj et al. used “sticky bone” (bone graft mixed with i-PRF) along with a collagen membrane for regeneration in an endo-perio case. The result showed a reduction in the probing depth by 5 mm and satisfactory bone formation at the end of 6 months [39]. Mila Vučković et al. completed a study to compare the regenerative effect of i-PRF when injected directly into a horizontal bone defect with the help of a stent in a chronic periodontitis patient with saline as the control. After 3 months, a better reduction in probing depth and gain in clinical attachment level was seen in the i-PRF group [40]. In the present study, better periodontal and bone regeneration was noted in the group with i-PRF and nano-HA bone grafts. It could be due to the fact that because of the slow centrifugation speed and the less time taken for the preparation of i-PRF, probably more amount of growth factors may be retained in the top layer above the buffy coat. Along with the growth factors, a number of leucocytes may also be present in the i-PRF which might have an anti-inflammatory effect [41] thus aiding in better healing of the tissues. (Abd El Raouf et al., 2019) [42] Along with nano-HA bone graft, i-PRF can have an osteoinductive effect on the neighbouring cells due to the release of growth factors and bone morphogenetic proteins [43,44]. Hence, the nano-HA crystalline bone graft showed better comparable results when used with i-PRF.

The limitations of the study included the small sample size, no post-operative follow-up and no histological determination of the new bone regeneration. Within these limitations, the results of this study suggested that a nano-hydroxyapatite bone graft (Sybograft, Eucare pharmaceutical pvt LTD. Chennai, India) material is quite predictable when used with i-PRF in the treatment of Grade II furcation defects in mandibular molars in bringing about bone fill and improvement in clinical parameters. However, further investigation with a larger sample size on a prolonged post-operative follow-up is required to conclusively establish the outcome of this study. Further, histological evaluation to determine other changes such as the type of new bone that is formed, any evidence of cementogenesis and regeneration of the attachment apparatus should be taken up in future studies.

## 5. Conclusions

The study looked at the regenerative adequacy of i-PRF with bone graft in the treatment of mandibular grade II furcation. The outcomes showed a greater clinical as well as radiographical improvement in the two groups; however, bone fill in the I-PRF with bone graft showed better outcomes. Hence, i-PRF can be viewed as a promising material in advancing periodontal tissue regeneration.

## Figures and Tables

**Table 1:** Demographic Data.

Groups *	N	Mean	Std. Deviation	Median	Male	Female
I	12	37.5	9.62	34	6	6
II	12	33	4.20	33.5	6	6

* Group-I Control group; Group-II test group.

**Table 2:** Comparison of mean scores between test and control groups at baseline for various clinical parameters by independent *t*-test.

		N	Mean	SD	*p*-Value
**PI**	Baseline–Test	12	1.4050	0.2813	0.3899
	Baseline–Control	12	1.2917	0.1275	
**GI**	Baseline–Test	12	1.4550	0.2591	0.1595
	Baseline–Control	12	1.2717	0.1419	
**PPD**	Baseline–Test	12	6.0000	0.8944	0.4388
	Baseline–Control	12	5.5000	1.2247	
**CAL**	Baseline–Test	12	5.1667	0.7528	0.1306
	Baseline–Control	12	3.8333	1.8349	
**HPD**	Baseline–Test	12	4.0000	1.2649	0.6372
	Baseline–Control	12	4.3333	1.0328	
**VPD**	Baseline–Test	12	3.5000	0.5477	0.8339
	Baseline–Control	12	3.3333	0.8165	

Statistically significant at 5% level of significance.

**Table 3:** Comparison of mean clinical parameters scores of within groups at baseline, 3 months, 6 months and 9 months.

	Group	Mean	Std. Deviation	N	Sig.
PI_base	Test	1.4050	0.28126	12	0.334
	Control	1.2917	0.12750	12	
PI_3mon	Test	1.0400	0.27554	12	
	Control	1.0550	0.27304	12	
PI_6mon	Test	0.6783	0.22912	12	
	Control	0.8217	0.35005	12	
PI_9mon	Test	0.4400	0.16982	12	
	Control	0.5483	0.22807	12	
GI_base	Test	1.4550	0.25906	12	0.140
	Control	1.2717	0.14190	12	
GI_3mon	Test	1.0983	0.19062	12	
	Control	1.0433	0.27732	12	
GI_6mon	Test	0.7633	0.14348	12	
	Control	0.7950	0.32415	12	
GI_9mon	Test	0.3233	0.15042	12	
	Control	0.5783	0.32109	12	
PPD_base	Test	6.0000	0.89443	12	0.008 *
	Control	5.5000	1.22474	12	
PPD_3mon	Test	4.0000	1.09545	12	
	Control	3.8333	1.32916	12	
PPD_6mon	Test	2.0000	0.00000	12	
	Control	3.0000	0.89443	12	
PPD_9mon	Test	1.3333	0.51640	12	
	Control	2.1667	0.40825	12	
CAL_base	Test	5.1667	0.75277	12	0.001 *
	Control	3.8333	1.83485	12	
CAL_3mon	Test	3.1667	0.98319	12	
	Control	3.1667	1.32916	12	
CAL_6mon	Test	1.0000	0.00000	12	
	Control	2.3333	0.81650	12	
CAL_9mon	Test	1.0000	0.00000	12	
	Control	1.3333	0.51640	12	
HPD_base	Test	4.0000	1.26491	12	0.012 *
	Control	4.3333	1.03280	12	
HPD_3mon	Test	3.3333	1.50555	12	
	Control	4.0000	1.26491	12	
HPD_6mon	Test	1.3333	0.81650	12	
	Control	3.1667	1.32916	12	
HPD_9mon	Test	0.6667	0.51640	12	
	Control	2.6667	1.21106	12	
VPD_base	Test	3.5000	0.54772	12	0.000 *
	Control	3.3333	0.81650	12	
VPD_3mon	Test	3.1667	0.75277	12	
	Control	3.0000	1.09545	12	
VPD_6mon	Test	1.1667	0.40825	12	
	Control	2.5000	0.83666	12	
VPD_9mon	Test	0.6667	0.51640	12	
	Control	2.0000	0.63246	12	
baf_base	Test	2.9167	0.88863	12	0.000 *
	Control	3.4000	1.39857	12	
baf_9m	Test	5.6833	1.10529	12	
	Control	3.9833	1.40060	12	

* Statistically significant at 5% level of significance.

## Data Availability

Data can be made available on demand by chief researcher for academic purpose by email.
